# Socioeconomic-related inequalities in child malnutrition: evidence from the Ghana multiple indicator cluster survey

**DOI:** 10.1186/s13561-015-0072-4

**Published:** 2015-11-24

**Authors:** Jacob Novignon, Emmanuel Aboagye, Otuo Serebour Agyemang, Genevieve Aryeetey

**Affiliations:** 1 Department of Economics, University of Ibadan, Ibadan, Nigeria; 2 Department of Health Economics, Policy and Management, University of Oslo, Oslo, Norway; 3 School of Business, University of Cape Coast, Cape Coast, Ghana; 4 Department of Health Policy, Planning and Management, School of Public Health, University of Ghana, Legon, Ghana

**Keywords:** Child malnutrition, Inequality, Concentration index, Mother’s education, Health insurance

## Abstract

**Background:**

Malnutrition is a prevalent public health concern in Ghana. While studies have identified factors that influence child malnutrition and related inequalities in Ghana, very little efforts have been made to decompose these inequalities across various household characteristics. This study examined the influence of socioeconomic factors on inequality in child malnutrition using a decomposition approach.

**Methods:**

The study employed cross section data from the 2011 Multiple Indicator Cluster Survey (MICS). Analysis was done at three levels: First, concentration curves were constructed to explore the nature of inequality in child malnutrition. Secondly, concentration indices were computed to quantify the magnitude of inequality. Thirdly, decomposition analysis was conducted to determine the role of mother’s education and health insurance coverage in inequality of child malnutrition.

**Results:**

The concentration curves showed that there exists a pro-poor inequality in child malnutrition measured by stunting and wasting. The concentration indices of these measures indicated that the magnitude of inequality was higher and significant at 1 % for weight-for-age (WAZ) (−0.1641), relative to height-for-age (HAZ) (−0.1613). The decomposition analyses show that whilst mother’s education contributed about 13 and 11 % to inequality in HAZ, it contributed about 18.9 and 11.8 % to inequality in WAZ for primary and secondary or above education attainments, respectively. Finally, health insurance contributed about 1.91 and 1.03 % to inequality in HAZ and WAZ, respectively.

**Conclusion:**

The results suggest that there is the need to encourage critical policies directed towards improving female literacy in the country. The existence of a functional health insurance system and increasing universal coverage are recommended to mitigate child malnutrition.

## Background

Child malnutrition is a prevalent public health concern in sub-Saharan Africa [1, 2]. In 2012, the number of children under age five in the region estimated to be malnourished was 21 and 38 % for weight-for-age and height-for-age scores, respectively [3, 4]. Child malnutrition could have implications for physical and cognitive growth of a child, decreased work ability in adult life thus causing loss in productivity [5, 6]. Child malnutrition is associated with morbidity and mortality among children, and also hampers their mental development, educational performance and intellect [7, 8].

Malnutrition is usually measured by impairment in growth in weight and height [9]. There is global acceptance that children have almost equal possibility of growth, before they attain their seventh year [10]. Thus socioeconomic and demographic factors seem to be more pertinent than hereditary features, in growth disparities among children [11]. There is considerable evidence in the health economics literature that suggest that child nutritional status is related to a number of socioeconomic factors such as household wealth, rural/urban residence, mothers’ education, demographic factors and access to health care services. For instance, some studies showed that demographic factors such as the child’s age, sex and birth size have been linked to child nutritional status [12–15]. Moreover, a number of studies have found consistent disparities in the prevalence of malnutrition along the lines of age, sex and birth size of children [16–19]. Also, there are consistent findings in child malnutrition studies that households’ wealth, usually measured by increments in household material standards (calculated into a wealth index) is associated with childhood nutritional status [20, 21]. It is argued that children from the poorest households are stunted or underweight compared to children from richest households [22].

Higher rates of stunting and underweight have been associated to children who reside in rural areas than those in urban areas [23, 24]. Also, the level of mother’s educational attainment is consistently associated with child malnutrition [25, 26]. Regarding access to health care services, it has been shown that children from households having difficulty in accessing health care services suffer from considerably higher levels of childhood malnutrition [27, 28].

Although the aforesaid factors have been argued to influence childhood nutritional status, most of these studies have examined only the direction of association with childhood nutritional status. In the case of Ghana, an important contribution will be to show how certain socioeconomic factors contribute to disparity in child malnutrition and how this contribution explains the burden of childhood malnutrition. Thus in order to appreciate the degree to which each socioeconomic factor contributes to this burden, the study examines the influence of these factors on inequalities in childhood malnutrition. The relevance of this approach is that it allows the recognition of the factors that contribute to socioeconomic-related inequality in childhood nutritional status. Though its importance has been verified on data from advanced countries, it has known meagre applications in developing countries particularly, sub-Saharan African countries.

Again, the current study adds to existing literature by including variables that are particularly crucial in such analysis in the case of Ghana. These variables are health insurance coverage and source of antenatal care (i.e. skilled/unskilled antenatal care). Controlling for this variables is important because Ghana has a fully functional health insurance scheme which also exempts pregnant women from paying for antenatal care. As mentioned earlier, health insurance coverage has the possibility of minimizing the difficulty in accessing healthcare services for children and thus the burden of childhood malnutrition.

## Methods

### Data and variables

Data used in the study was from the 2011 Multiple Indicator Cluster Survey (MICS). The data is the fourth in a series of nationally representative sample survey of households, children aged 0–5 years, women aged 15–49 years and men aged 15–59 years. The survey was conducted by the Ghana Statistical Service with financial and technical support from UNICEF, USAID/CDC, UNFPA, the Japanese Government and the Ministry of Health. The basic objective of the MICS was to monitor the health of mothers and children using various indicators. Information collected generally include nutrition, immunization coverage, health care utilization, health insurance coverage, socioeconomic characteristics, sexual behaviours etc. [29].

Both urban and rural areas for all ten regions of the country were included in the sample design. The urban and rural areas were identified as the main sampling strata with selection done in two stages. Within each stratum, enumeration areas were systematically selected with probability proportional to size.

Three different measures of child nutrition were used for the analysis. These were height-for-age (HAZ), weight-for-age (WAZ) and weight-for-height (WHZ) z-scores, based on the World Health Organization (WHO) definitions. The z-scores were further categorised into stunted, under-weight and wasting, respectively indicating growth retardation defined as HAZ, WAZ and WHZ below -2 z-scores. This is a standard cut-off point proposed by the WHO to measure malnutrition [30]. This limit also gives a good indication of increased risk of morbidity and mortality among children. Various individual characteristics that contribute to inequality in child malnutrition were also included in the analysis. These variables include; household economic status (measured by wealth quintiles based on household asset endowments), source of antenatal care, health insurance (if mother has valid insurance coverage or not), mother’s formal educational attainment, child size, place of residence (rural or urban), and sex of child (male or female) [29]. As mentioned earlier, the inclusion of health insurance coverage was important because of its relevance to health care access and hence improved malnutrition. It is expected that women with easy access to health facility will likely understand the nutritional needs of a child. Such lessons are also part of antenatal sessions.

### Univariate and bivariate analysis

The analysis starts with an analysis of the individual variables separately. The purpose of this was to examine the proportion of respondents with specific characteristics including the dependent variable, child malnutrition. Frequencies including the number of respondents with particular characteristics and corresponding percentages were used in the analysis. The bivariate analysis used frequencies and percentages from cross-tabulations to analyse the association between the dependent variable and socioeconomic characteristics of respondents. Chi-square tests were computed to verify the significance of association.

### Econometric analysis

The econometric analysis was performed at three levels; first concentration curves for the child malnutrition variables were constructed. Second, the concentration indices for these variables were computed to augment the concentration curves. The final analysis was to decompose the concentration indices to identify factors that contribute to inequality in child malnutrition.

The concentration curve gives a pictorial view of the pattern and magnitude of inequality in child malnutrition. The curve is a plot of the cumulative percentage of child malnutrition on the y-axis and wealth status ranked by cumulative percentage of the population on the x-axis. The concentration curves depict inequality against the poor if it lies above the line of equality (45^0^ line). On the other hand, inequality against the rich exists if the curve lies below the line of equality. In a situation where there exists perfect equality in child malnutrition, irrespective of wealth status, the concentration curve is a straight line equal to the 45^0^ line. The magnitude of inequality is depicted by how far the curve lies away from the line of equality. For instance, if the magnitude of inequality in favour of the rich is higher, the farther the curve will be above the line of equality.

To ascertain the magnitude of and nature of socioeconomic-related inequality in child malnutrition, the concentration indices (CI) were computed. This approach of measuring inequality has been widely used and recognized as a standard tool. The index is defined as “twice the area between the concentration curve and the line of equality” [31]. The CI can be computed as the covariance between child malnutrition and the fractional rank in wealth score distribution;

1 $$ C=\frac{2}{u}\operatorname{cov}\left({y}_{{}_i},{r}_i\right) $$

where *C* is concentration index, *y*_*i*_ is child malnutrition, *r*_*i*_ is the fractional rank of individual *i* in the distribution of wealth score. *u* is the mean of the child malnutrition variable and *cov* is covariance.

The concentration index (CI) can either be positive or negative. As noted by [31], the sign of the CI gives an idea of the direction of relationship between child malnutrition variable and position in the wealth score distribution. A negative sign shows inequality concentrated among the poor population while a positive sign suggests inequality in favour of the rich. In the absence of inequality (perfect equality), the CI is zero. The value of the CI ranges between−1 and +1 (i.e.,−1 ≤ CI ≤ 1) and the magnitude of the CI value provides information about the strength of the relationship and the degree of variability in child malnutrition. The closer the absolute value of the CI to one, the higher/stronger the level of inequality.

To understand the contribution of individual socioeconomic characteristics to inequality in childhood malnutrition, the estimated CIs were decomposed. Following O’Donnell et al. [31] and Wagstaff et al. [32], the contribution of each individual characteristic is defined as the product of the sensitivity of health with respect to that characteristic and the degree of inequality in that factor. Therefore, suppose a linear addition regression model of child malnutrition (*y*), given as

2 $$ y=\alpha +{\displaystyle {\sum}_k{\beta}_k{x}_k+\varepsilon } $$

we can write the CI, c, for y as

3 $$ c={\displaystyle {\sum}_k\left({\beta}_k{\overline{x}}_k/\mu \right)}{c}_k+G{C}_{\varepsilon }/\mu $$

where μ is mean of *y*, ${\overline{x}}_k$ is mean of *x*_*k*_ *, C*_*k*_ is a CI for *x*_*k*_ and GC_ε_ is the generalized CI for the error term (ε). Two relevant components of Equation (3) can be distinguished; (1) the first term on the right hand side of the equation shows a weighted sum of the CI of *k* regressions, where the weight ${\overline{x}}_k$ is the elasticity of *y* with respect to *x*_*k*_ $\left({\eta}_k={\beta}_k\frac{{\overline{x}}_k}{\mu}\right)$ (2) the second term on the right hand side is the residual component which shows unexplained inequality.

O’Donnell et al. [31] noted that since the CI may inevitably lose some information contained in the concentration curve from which it was computed, it is critical for the index to be examined in conjunction with the curves. In this regard, the concentration curves for the respective child malnutrition measures were complemented with the CIs. The statistical significance of the CIs was also computed using a bootstrap procedure. Variables that showed potential multicollinearity were dropped from the analysis.

## Results

### Descriptive statistics (Univariate analysis)

Table 1 provides descriptive statistics for the variables included in the analysis. Means, numbers (with percentages in parenthesis), minimum and maximum values were reported. The table shows on average, height-for-age, weight-for-age and weight-for-age z-scores of approximately 1.24, 1.00 and 0.41, respectively. There was significantly higher proportion of respondents in rural (71.9 %) than urban (28.2 %) areas. About 53.8 % of mothers did not have any formal education, significantly higher than those with at least secondary level education (7.1 %). Similarly, about 46.5 % of respondents were in the lowest wealth quintile, relative to 8.4 % in the highest quintile. The statistics also showed that there were more male children than female children. About 54.5 % of the sample were covered by health insurance, relative to 45.5 % without health insurance coverage. Significant proportion (96.1 %) of pregnant women who sought antenatal care did so from skilled attendants.

**Table 1:** Descriptive statistics

Variable	Mean	Number(%)	Minimum	Maximum
Child health outcomes				
HAZ	1.246368		−5.94	5.95
WAZ	0.996846		−3.52	5.69
WHZ	0.411732		−5	4.97
Place of residence				
Urban		2023 (28.15)	0	1
Rural		5163 (71.85)	0	1
Mother’s education				
None		3863 (53.83)	0	1
Primary		2802 (39.05)	0	1
Secondary +		511 (7.12)	0	1
Wealth quintile				
Poorest		3337 (46.50)	0	1
Poor		1438 (20.04)	0	1
Middle		995 (13.87)	0	1
Rich		803 (11.19)	0	1
Richest		603 (8.40)	0	1
Sex of child				
Male		3666 (51.09)	0	1
Female		3510 (48.91)	0	1
Administrative region				
Western		379 (5.28)	0	1
Central		967 (13.48)	0	1
Greater Accra		375 (5.23)	0	1
Volta		378 (5.27)	0	1
Eastern		331 (4.61)	0	1
Ashanti		448 (6.24)	0	1
Brong Ahafo		384 (5.35)	0	1
Nothern		1904 (26.53)	0	1
Upper East		944 (13.15)	0	1
Upper West		1066 (14.86)	0	1
Child size at birth				
Very large		762 (41.23)	0	1
Larger than average		837 (45.29)	0	1
Average		249 (13.47)	0	1
Health insurance coverage				
Yes		3909 (54.47 %)	0	1
No		3267 (45.53 %)	0	1
Antenatal care				
Skilled		1720 (96.09 %)	0	1
Unskilled		70 (3.91 %)	0	1

Source: Authors’ computation

### Bivariate analysis

Table 2 shows results for bivariate analysis of association between socioeconomic characteristics and child malnutrition. Child malnutrition was measured by the separate binary variables, namely; whether a child was stunted, underweight or wasted.

**Table 2:** Bivariate analysis

Variables	Stunted *N* = 1934	Chi-Square	Under weight *N* = 1200	Chi-square	Wasted *N* = 541	Chi-square
Place of residence						
Urban	418 (21.3)	61.3911***	254 (20.89)	37.5728***	130 (23.85)	5.3729**
Rural	1544 (71.90)		962 (79.11)		415 (76.15)	
Mother’s education						
None	1257 (64.07)	125.7010***	788(64.91)	71.9399***	331 (60.96)	17.6267***
Primary	631 (32.16)		367(30.23)		173 (31.86)	
Secondary +	74 (3.77)		59(4.86)		39 (7.18)	
Wealth quintile						
Poorest	1099 (56.01)	179.0416***	707 (58.14)	113.2899***	289 (53.03)	28.6886***
Poor	414 (21.10)		241 (19.82)		108 (19.82)	
Middle	248 (12.64)		134 (11.02)		71 (13.03)	
Rich	130 (6.63)		96 (7.89)		47 (8.62)	
Richest	71 (3.62)		38 (3.13)		30 (5.50)	
Sex of child						
Male	1093 (55.71)	23.3813***	679 (55.84)	13.4240***	335 (61.47)	28.1695***
Female	869 (44.29)		537 (44.16)		70 (57.38)	
Administrative region						
Western	90 (4.59)	192.5907***	55 (4.52)	154.9754***	31 (5.69)	60.7955***
Central	234 (11.93)		134 (11.02)		60 (11.01)	
Greater Accra	46 (2.34)		28 (2.30)		14 (2.57)	
Volta	77 (3.92)		43 (3.54)		31 (5.69)	
Eastern	77 (3.92)		38 (3.13)		23 (4.22)	
Ashanti	103 (5.25)		57 (4.69)		30 (5.50)	
Brong Ahafo	70 (3.57)		38 (3.13)		12 (2.20)	
Northern	716 (36.49)		471 (38.73)		167 (30.64)	
Upper East	288 (14.68)		185 (15.21)		75 (13.76)	
Upper West	261 (13.30)		167 (13.73)		102 (18.72)	
Child size at birth						
Very large	209 (40.90)	0.3193	140 (44.59)	2.1032	61 (44.53)	7.5255
Above average	230 (45.01)		130 (41.40)		63 (45.99)	
Average	72 (14.09)		44 (14.01)		13 (9.49)	
Health insurance						
Yes	999 (51.65 %)	8.4815***	586 (48.83 %)	18.4819***	253 (46.77 %)	16.8824***
No	935 (48.35)		614 (51.17 %)		288 (53.23 %)	
Antenatal care						
Skilled	466 (95.88 %)	0.0743	285 (95.00 %)	1.1382	129 (98.47 %)	2.2200
Unskilled	20 (4.12 %)		15 (5.00 %)		2 (1.53 %)	

Source: Authors’ computation

Statistical significance were marked as **p < 0.05, ***p < 0.01. Percentages are reported in parenthesis

The results show that child malnutrition was prevalent in rural areas compared to urban areas. About 71.9 % of children who were stunted were from rural communities. Similarly, about 79.1 % of under-weight children and 76.2 % of wasted children were from rural areas. Growth retardation among children reduced with higher education attainments of their mothers. Majority of children whose mothers have no formal education were stunted (64.1 %), under-weight (64.9 %) and wasted (61.0 %), relative to children whose mothers had secondary or higher education attainments. Similar association was established between household wealth status and child malnutrition. The proportion of children from households in the richest wealth quintile and suffering from stunting was 3.6 %, under-weight was 3.1 % while wasted was 5.5 %. Child malnutrition was also higher among male children relative to their female counterparts. Child malnutrition was also highest in the three northern regions (namely; Northern, Upper East and Upper West regions). The Greater Accra region recorded the lowest child malnutrition in Ghana.

### Inequality in child malnutrition

**Fig. 1:**
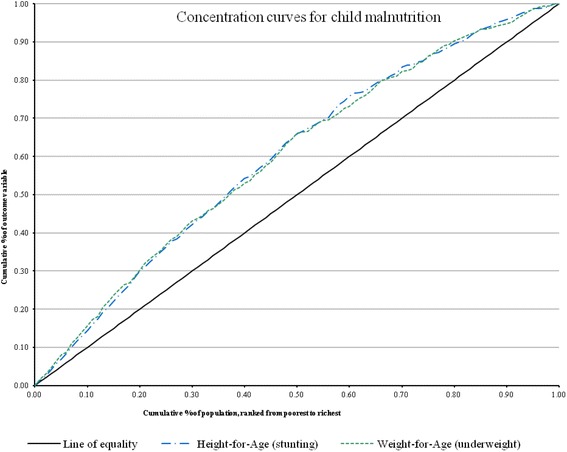
Concentration curves

The concentration curves in Fig. 1 show that inequality in child growth retardation or malnutrition was concentrated among the poor. Both concentration curves lie above the line of equality which implies that child malnutrition is concentrated among children from poor households. A close investigation of the individual curves shows that there is no clear difference, in terms of magnitude, between the specific measures of child malnutrition. However, the concentration indices of these measures indicate that the magnitude of inequality was higher for weight-for-age (under-weight), relative to height-for-age (stunting). Concentration indices of about−0.1641 and−0.1613 were estimated for weight-for-age and height-for-age child malnutrition measures, respectively. The sign of the concentration indices complements the findings of the concentration curves that child malnutrition was concentrated among the poor.

### Decomposing inequality in child malnutrition

Tables 3 and 4 present results from the decomposition analysis which shows how the various socioeconomic characteristics of respondents contribute to inequality in child malnutrition. Three different analysis were conducted for the decomposition results. First, the impact of the various individual characteristics on child malnutrition was estimated. Secondly, the concentration index for each of the socioeconomic variables was computed to show how unequal a particular variable was distributed. Finally, the percentage contribution of each of the variables to inequality in child malnutrition was computed.

**Table 3:** Decomposition results from height-for-age scores

Variables	Estimated Coefficients	Concentration Index	Percentage Contribution
Place of residence (urban)	−0.0732	0.56550***	7.85282
	(0.0551)	(0.0153)	(5.2993)
Mother’s education attainment			
Primary	−0.17636***	0.27811***	12.91317***
	(0.0508)	(0.0117)	(3.3894)
Secondary and above	−0.30692***	0.71319***	10.50996**
	(0.1155)	(0.0195)	(4.0899)
Source of antenatal care			
Doctor	−0.39623**	0.13920***	1.41282*
	(0.1584)	(0.0519)	(0.7509)
Nurse/Midwife	−0.19795*	−0.01113***	−1.37141*
	(0.1040)	(0.0033)	(0.7924)
Health insurance	−0.10924*	0.04766***	1.91208***
	(0.0589)	(0.0109)	(0.6514)
Age of child	0.21001***	−0.01097	3.13153
	(0.0188)	(0.0073)	(1.9676)
Child size at birth			
Very large	−0.0189	0.00012	0.00065
	(0.0709)	(0.0135)	(0.2493)
Larger than average	−0.05438	0.00397	0.06592
	(0.0666)	(0.0118)	(0.3196)
Household wealth quintile			
Middle	−0.15129**	0.46955***	6.64145**
	(0.0766)	(0.0109)	(3.2391)
Rich	−0.46485***	0.80418***	49.38621***
	(0.0726)	(0.0105)	(6.3955)

Source: Authors’ computation

Statistical significance were marked as * =*p* < 0.10, ** =*p* < 0.05, *** =*p* < 0.01. Bootstrapped standard errors are reported in parenthesis

**Table 4:** Decomposition results for weight-for-age scores

Variables	Estimated Coefficients	Concentration Index	Percentage Contribution
Place of residence (urban)	−0.10598**	0.56550***	14.35321**
	(0.0484)	(0.0153)	(5.7306)
Mother’s education attainment			
Primary	−0.20461***	0.27811***	18.91250***
	(0.0432)	(0.0117)	(3.5619)
Secondary and above	−0.27326***	0.71319***	11.81246***
	(0.0797)	(0.0195)	(3.5961)
Source of antenatal care			
Doctor	0.04265	0.13920***	−0.19195
	(0.1286)	(0.0519)	(0.6896)
Nurse/Midwife	−0.03193	−0.01113***	−0.27925
	(0.0864)	(0.0033)	(0.7374)
Health insurance	−0.04665	0.04766***	1.03069*
	(0.0392)	(0.0109)	(0.5464)
Age of child	0.06196***	−0.01097	1.16626
	(0.0133)	(0.0073)	(0.7819)
Child size at birth			
Very large	0.00318	0.00012	−0.00014
	(0.0528)	(0.0135)	(0.1967)
Larger than average	−0.06838	0.00397	0.10462
	(0.0559)	(0.0118)	(0.4246)
Household wealth quintile			
Middle	−0.07669	0.46955***	4.24967
	(0.0629)	(0.0109)	(3.1679)
Rich	−0.31811***	0.80418***	42.66306***
	(0.0692)	(0.0105)	(7.0602)

Source: Authors’ computation

Statistical significance were marked as * = p < 0.10, ** =*p* < 0.05, ***=*p* < 0.01. Bootstrapped standard errors are reported in parenthesis

The estimated coefficients suggest that mother’s education, source of antenatal care, health insurance and household wealth status were significant contributors to child malnutrition. Table 3 shows that, relative to no formal education, children whose mothers had primary or secondary and above educational attainments were more likely to have better height-for-age score. Similar relationship was established for the weight-for-age regression in Table 4. Children whose mothers sought for antenatal care from doctors or nurses were more likely to also report higher height-for-age scores, relative to those who sought antenatal care from other sources such as traditional birth attendants. The relationship was, however, not significant in the weight-for-age regression. Relative to children from households in the poor wealth quintile, children from rich quintile were less likely to report lower values of height-for-age and weight-for-age.

The concentration indices for the explanatory variables suggest that with the exception of seeking antenatal care from nurses/midwives and child age, inequality for all the other variables was concentrated among the rich. For instance, urban residence, higher maternal education, seeking antenatal care from doctors and having health insurance showed positive and significant sign, indicating pro-rich inequality.

The percentage contribution of the various explanatory variables to inequality in child malnutrition is presented in the last column of Tables 3 and 4. While the absolute values show the extent to which a particular variable contributes to inequality, a positive (negative) sign is an indication that the variable contributes (does not contribute) to inequality.

Results from the height-for-age score indicate that mother’s education, sourcing antenatal care from a doctor, having health insurance coverage and wealth status play important roles in inequality in child malnutrition. In the case of results from the weight-for-age measure of child malnutrition, mother’s education, health insurance and household wealth status were important for child malnutrition. Specifically, households in the highest wealth quintile contribute about 49 % of total inequality in child malnutrition measured by height-for-age. Another major contributor was mother’s education which contributed about 13 and 11 % for primary and above secondary attainments, respectively (Table 3). In Table 4, belonging to a household in the highest wealth quintile contributed about 42.6 % to child malnutrition. Also, primary and secondary or above education of mothers contributed about 18.9 and 11.8 %, respectively, to inequality in child malnutrition measured by weight-for-age. Sourcing antenatal care from nurses/midwives was the only less important contributor to inequality in child malnutrition in both Tables 3 and 4.

## Discussion

The results of the study show that there is statistically significant differences in child malnutrition by place of residence (with rural accounting for more than 70 % of malnourished children), mother’s education level (mothers with no education accounting for more than 60 % of malnourished), wealth status (with poorest/lowest quintile accounting for more than 53 % of malnourished children), sex of child (females more than 55 % of malnourished) and region (with Northern region accounting for more than 30 % and Greater Accra Region less than 3 % of malnourished). These differences hold for each category of malnutrition used in the bivariate analysis (i.e. stunting, under-weight and wasting) and are significant at 1 % (except for rural–urban under wasting, which was significant at 5 %).

The result of differences in child malnutrition by location and mother’s education conforms with the well-known, long-standing problem that has been reported in other studies in developing countries such as Mozambique and Uganda ([23–26]). Backed by the World Health Organization and UNICEF, the Community-based Management of Acute Malnutrition (CMAM) programme was initiated in 2007 and implemented in developing countries such as Ghana, Ethiopia, Malawi, Zambia, Indonesia and Bangladesh [33] to improve access to health care for malnourished children across the geographic and socioeconomic divide, thereby improving health outcomes. However, though the programme may have been successful in parts of the country (i.e. Ghana), other parts of the country may not have been covered. An important way of bridging the rural–urban gap with respect to child malnutrition is to strengthen and make use of existing close-to-client health care arrangements such as the Community-based Health Planning and Services initiative and the use of community health volunteers to reduce child malnutrition. It is important to state that some of the categories reported above may be linked to each other. For instance, rural areas tend to have a large proportion of uneducated mothers, those with low wealth and found mostly in the regions that reported high cases of malnutrition. Therefore, a concerted effort to approaching this will be to tackle not just the health aspect, but also poverty reduction through programmes that empower women and improve their access to education and resources as these would go a long way to reduce child malnutrition in these areas.

Sex-specific differences in child malnutrition revealed that there is a significant decline among males. This empirical result is also shown in [11] in their description of patterns in child malnutrition stratified by sex. The result may reflect mere differentials by sex as a demographic factor as would other socioeconomic factors such as maternal education, urban-rural differences etc., and not a deliberate effort by families to favour boys. Some of these significant improvements could be attributed to the introduction of nutrition rehabilitation services and Supplementary Feeding and Health and Nutrition Education Programme in deprived communities, mostly rural communities. This argument however is only speculative.

The concentration indices estimated were−0.1641 for weight-for-age and−0.1613 for height-for-age. These, together with the corresponding concentration curves, show that the poor continue to be disproportionately affected by underweight and stunting in Ghana, despite efforts to curb and neutralise the socioeconomic disparities in child malnutrition. This evidence from very recent data confirms the finding of van de Poel et al. – using 2004 data – that stunting and wasting affected the poor disproportionately. Thus, about a decade after van de Poel’s finding, not much has changed with respect to socioeconomic inequality in child malnutrition in Ghana, though our finding on stunting represents slight reduction from van de Poel’s finding of−0.19. There is the need for rapid scale up of investments in important, innovative and cost-effective interventions (such as the CMAM) that target child malnutrition while closing the socioeconomic gap [34]. The methodological significance of this study is that the concentration curves for stunting and underweight show similar patterns and indices that are close, indicating that these two measures of malnutrition could be used alternatively with little differences. This is in contrast to van de Poel’s study which found significant differences between the concentration indices for stunting (−0.19) and wasting (−0.00).

Mother’s education level, child’s age, source of antenatal care (i.e. skilled birth attendance), health insurance coverage and household wealth status were significantly associated with height-for-age while place of residence, mother’s education, child’s age and wealth status were significantly associated with weight-for-age in Ghanaian children. Most of these results confirm the findings of other studies [6, 13, 23, 25] with the exception of child size at birth in relation to height-for-age and weight-for-age scores. The other factors (i.e. source of antenatal care and health insurance coverage) we have endogenously examined were also significant and associated with weight-for-age and height-for-age scores, respectively.

The findings of this study further show that for both height-for-age and weight-for-age, wealth status was the largest contributor to inequality, contributing about 49 % to inequality in stunting and 43 % to underweight. Other important contributors to inequality include mother’s education level and rural–urban location. Undoubtedly, these variables have remained important factors to consider in designing policies to reduce malnutrition in developing countries like Ghana. Thus, the effect of policies targeting reduction of rural–urban differences in development and health care provision, and improving literacy and empowerment of women and girls (including girl child education) could be gauged from the rate of malnutrition in children. Not surprisingly, health insurance coverage contributed to inequality and this can largely be attributed to the various exemptions available for mothers and children under age 18. Exemptions for mothers and children were geared towards improving equity in access to health care. The exemption was for all maternal healthcare services including caesarean deliveries and emergency care. The principal objective was to reduce maternal and child mortality as well as improving their health status. Such selective free care was also extended to the aged population (above age 65). This easy access to health care helps in early detection and treatment of malnutrition among children without any financial constraint. This is also relevant because before the introduction of this policy vulnerable and deprived populations were unable to easily access health care even though the suffered most from childhood malnutrition.

There are some limitations in this study. Firstly, this study shows factors that are associated with child malnutrition, socioeconomic inequality in malnutrition and the magnitude of the associations but no causal interpretation of the results is implied here. Secondly, maternal education, household wealth and place of residence were contributing to socioeconomic inequality. However, distinguishing between rural and urban areas might be a problem within the context of the paper, because big cities have heterogeneous groups with regard to these variables. However, there was no such data available to enable us to identify such variability in the urban areas. The study also assumed a linear additive relationship between the explanatory and dependent variable. The possibility of a non-linear relationship was not explored here. Finally, the wealth status measure was only based on household asset endowments.

## Conclusion

The study set out to understand the nature of socio-economic related inequality in childhood malnutrition and to decompose the contribution of various factors to this inequality. Child malnutrition status was measured by height-for-age, weight-for-age and weight-for-height. These were further categorised into dummy variables to measure child stunting, underweight and wasting. Using data from the 2011 Ghana MICS, analysis were conducted using cross-tabulations with chi square tests, concentration curves and concentration indices.

The findings suggest that there is statistically significant inequality in childhood malnutrition against the poor, irrespective of the measure of childhood nutritional status employed in the analysis. The findings also showed that individual socio-economic characteristics such as wealth status maternal education, place of residence, source of antenatal care and health insurance coverage are important contributors to improving inequality in childhood malnutrition.

Following the findings of the study, critical policy insights were deduced, including bridging the rural–urban development gap as well as enhancing literacy and empowerment of women and girls. It is important to note that, policies towards addressing rural–urban differences need to take cognisance of significant poverty levels that may exist in urban areas, especially in developing countries such as Ghana. The National Health Insurance Scheme has contributed positively to general inequality in health care access and utilization in Ghana, however increasing coverage, especially for maternal and child health is relevant. This can be achieved by extending exemption to cover mothers beyond six weeks after birth. There is also need for enough public education to encourage children and mothers, especially in rural communities, to take advantage of exemptions of the NHIS. This is particularly important because, while the selective free care discussed earlier has improved maternal and child healthcare access, a majority of the population are still not covered. Public sensitization will therefore be relevant in ensuring that every mother and child will be covered under this exemption policy.
